# Study of the Influence of the Backplate Position on EMAT Thickness-Measurement Signals

**DOI:** 10.3390/s22228741

**Published:** 2022-11-12

**Authors:** Junjie Wang, Xinjun Wu, Yun Song, Lingsi Sun

**Affiliations:** School of Mechanical Science & Engineering, Huazhong University of Science and Technology, Wuhan 430074, China

**Keywords:** damp coefficient, electromagnetic acoustic transducer, impedance, pulsed width, backplate

## Abstract

Nondestructive testing (NDT) is an essential method for assessing structural integrity in the oil and gas industry. Electromagnetic acoustic transducers (EMATs) have been extensively used to detect the wall-thickness reduction of plate-like structures, because they do not require direct contact. The pulse intervals of echoes are used to calculate the remnant thickness of structures. If the width of a single pulse is too large, multiple pulses will be superimposed, making it more difficult to extract the pulse interval. Thus, the width of a single pulse affects the resolution of measurements. This paper investigates the impacts of the backplate position on the pulse width and amplitude of thickness-measurement signals, using EMATs. By means of impedance modeling and measurement, it can be shown that the output impedance of the receiving coil is strongly influenced by the coil-backplate gap. With the increment in the coil-backplate gap, the signal amplitude and damping coefficient increase, while the self-resonant frequency decreases. By means of signal measurements on the specimen, it is shown that the pulse width and the signal amplitude can be significantly influenced by the backplate position. By reducing the coil–backplate gap, the pulse width can be reduced by over 80%, and by increasing the gap, the signal amplitude can be increased by over 300%. These research results can be used to optimize EMAT design, thereby suppressing the superposition of pulse echoes.

## 1. Introduction

In the oil and gas industry, metallic structural parts are susceptible to corrosion and wall-thickness reduction arising from the harsh environment [1,2]. Nondestructive testing (NDT) is an essential method for assessing structural integrity [3]. As one of the NDT methods, electromagnetic acoustic transducer (EMAT) technology can be used to detect the remnant thickness of structures [4,5]. EMAT is a kind of ultrasonic transducer, which measures thickness by the speed of sound and the pulsed echo interval. Hence, the characteristics of pulsed echo are essential, because it is directly related to the extraction of the pulse intervals and pulse width [6].

Generally, the oscillations in the ultrasonic transducers are underdamped [7]. However, the underdamped oscillations require a period of time to decay to lower amplitudes, which can cause the superposition of multiple echoes and reduce the resolution of a transducer [8]. In piezoelectric ultrasound transducers, backing materials can alleviate this problem. There are a number of studies pointing toward the backplate of the piezoelectric transducer [6,9,10,11]. Studies show that the waveform can be damped by attaching an acoustically absorbing material onto the back of the transducer, capable of absorbing and consuming energy in piezoelectric elements [8]. Several studies into piezoelectric ultrasonic transducers suggest that backplates of specific materials can be used to accelerate acoustic energy loss [10,11], suppress echoes in the transducer [6], and thus improve reception sensitivity [12]. The EMAT transducer operates by means of electromagnetic coupling, and cannot absorb mechanical vibrations in piezoelectric elements using a backplate. However, the backplate is also essential, as it can shield the noise interference in the magnets. Therefore, the backplate in the EMAT is a conductive metallic material, rather than an acoustically absorbent material, and it is often set between the magnet and the coil [4,13]. There have been various types of studies into backplate design in EMATs. A high-electrical-conductivity backplate can shield the sound waves in the magnet of a transducer [14]. The ferrite backplate can effectively enhance the eddy current and Lorentz force in a specimen, compared with the case of no backplate [13]. The silicon-steel-laminations backplate can increase the eddy-current density and the dynamic magnetic-flux density in the specimen, enhancing the efficiency of the transducer [5]. Furthermore, the backplate can rearrange the current-source density across the coil conductor’s cross-sectional area, enhancing the lift-off performance [14,15,16]. To sum up, studies regard the backplate in the EMAT mainly focusing on the suppression of acoustic noise, the enhancement of amplitude, and the improvement of transducing efficiency. At the same time, these research studies mainly focus on the influence of backplates on the transmitting process of the EMAT. However, for the receiving process of the EMAT, the effect of backplates on the rate of energy loss has not received as much attention as the research field of the piezoelectric ultrasonic transducer.

The alternating current is present in the receiving coil during the reception process. The backplate is positioned just above the receiving coil in the EMAT, hence, the eddy currents at the same time are generated in the backplate, and it will also act on the receiving coil. For the receiving coils, the signal amplitudes of the EMATs can be promoted by employing coils with many turns [17]. Nevertheless, a practical coil contains self-capacitance and resistance, as well as pure inductance [18,19], and other signal characteristics may be changed in addition to the amplitude. In the equivalent model of a coil, the parameters of the equivalent elements depend not only on the coil structure, but also on the lift-off between the coil and the metallic specimens [13,18,19]. Furthermore, the impulse response of the equivalent circuit will be variable [20]. This means that when using coils, it is required to consider their parasitic parameters and the metallic conductors in the vicinity. Therefore, the backplate in the EMAT impacts the coil’s performance when it is located near the coil.

In line with the above statement, this paper investigates the relationship between backplate position and EMAT signal characteristics, which can be used to adjust the pulse signal’s width and amplitude. The structure of this paper is organized as follows. In Section 2, the receiving process of the EMAT is briefly analyzed using simulation, followed by the establishment of the equivalent model of the receiving coil and backplate. According to the equivalent model, the amplitude and width of a pulsed echo can be predicted by the variations of the equivalent inductance and the damping coefficient of the equivalent circuit. In Section 3, the impedances of the receiving coil–backplate structure with different gaps are obtained by impedance measurement, and an experiment on the specimen obtains the signals of EMATs. In Section 4, the parameters of equivalent elements are obtained by means of impedance-curve-fittings, followed by the verification of the equivalent circuit, using EMAT signals. Finally, the findings are summarized in Section 5.

## 2. Modeling of Receiving Process

### 2.1. Configuration of EMATs and Equivalent Model of Receiving Process

**Figure 1:**
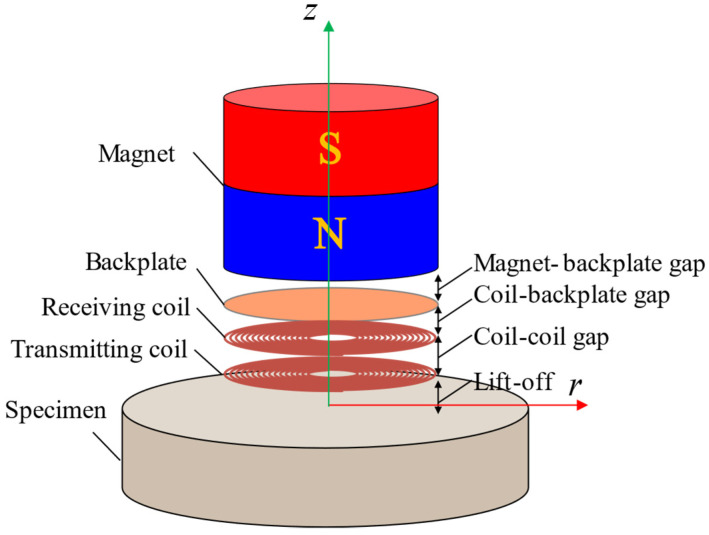
Schematic diagram of the electromagnetic acoustic transducer structure.

Figure 1 shows the configuration of the EMAT applied to thickness measurement. EMATs rely on the interaction of the magnetic-bias-field and the eddy currents to generate or receive elastic waves in the metallic structures [17,18,19].

The permanent magnet is used to produce a static magnetic-bias-field; the transmitting coil is used to produce eddy currents in a specimen; the receiving coil is capable of transducing the echoes, and is made in the form of a printed circuit board (PCB). The copper backplate is placed between the magnet and the receiving coil, and its position affects the performance of the receiving coil. Hence, this research mainly focuses on the gap between the backplate and the receiving coil. In order to distinguish between several gaps in the EMAT design, the lift-off is defined as the distance from the coil to the sample; the coil–coil gap is defined as the distance from the transmitting coil to the receiving coil, and the coil–backplate gap is defined as the distance from the receiving coil to the backplate; the magnet–backplate gap is defined as the distance from the magnet to the backplate.

In the EMAT reception process, the eddy currents due to the acoustic wave in the skin depth of a specimen are coupled by the receiving coil, creating an induced input current. Consequently, an alternating magnetic field will be generated around the receiving coil. Because the backplate is a metallic material with high electrical conductivity, the induced eddy current will be formed on its surface.

**Figure 2:**
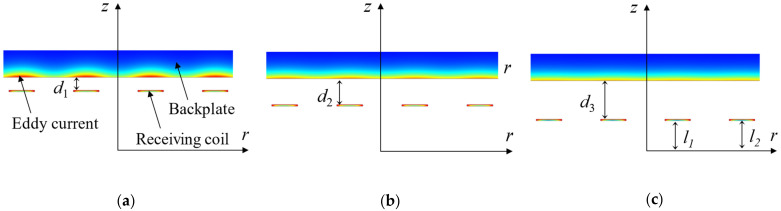
Section views of the eddy-current distribution when receiving coil–backplate gap, showing (**a**) *d*_1_, (**b**) *d*_2_ and (**c**) *d*_3_ separately, of which, *d*_1_ < *d*_2_ < *d*_3_.

According to Ampère’s circuital theorem in Equation (1), the eddy currents are also generated in the backplate above the coils, due to the change in magnetic flux. As is shown in Figure 2, these eddy currents are more significant when the gap between the coil and the backplate is smaller. According to Equations (2)–(5) [13,21], the eddy currents in the backplate are related to the position (ln) of the coil. Eventually, according to Equations (6) and (7) [13,21], the eddy-current impedance of the receiving coil would be influenced by the position of the backplate. The pulsed echo is acquired from the output of the receiving coil. Hence, the characteristics of the signal will be affected by the position of the backplate, due to the change in coil impedance.

(1) $$\nabla \times \vec{B}=\mu_{0}{\vec{J}}_{coil}$$

(2) $$\nabla \times \vec{E}=-\frac{\partial \vec{B}}{\partial t}$$

(3) $$\vec{B}=\nabla \times \vec{A}$$

(4) $$\vec{E}=-\frac{\partial \vec{A}}{\partial t}$$

where $\vec{B}$ is the magnetic flux, *μ*_0_ is the permeability in a vacuum, $\vec{J}$ is the eddy-current density in the receiving coil, $\vec{A}$ is the magnetic vector potential, $\vec{E}$ is the induced electromotive force, and *t* is the time.

(5) $$\vec{J}=\sigma \vec{E}=-\sigma \frac{\partial \vec{A}}{\partial t}=-j\omega \sigma \vec{A}\left(r,z\right)$$

where $\vec{J}$ is the eddy-current density in the backplate, *σ* is the conductivity of the backplate, and *ω* is the angular frequency of current.

(6) $$V=j2\pi \omega \sum_{n=1}^{N}r_{n}A\left(r_{n},l_{n}\right)$$

where *r_n_* and *l_n_* represent the coordinates of the nth turn of the single-turn coil, respectively, and N represents the number of turns of the coil, when the same layer of the planar coil *l_n_* is a constant value. Specifically for the receiving coil, *r_n_* is the radius of each turn and all *l_n_* are the same, and equal to the gap between the receiving coil and the specimen.

(7) $$Z=\frac{V}{I_{c}}$$

where *Z* is the impedance produced by the eddy currents, *V* is the total voltage induced into a receiving coil, and *I* is the input current in a receiving coil.

**Figure 3:**
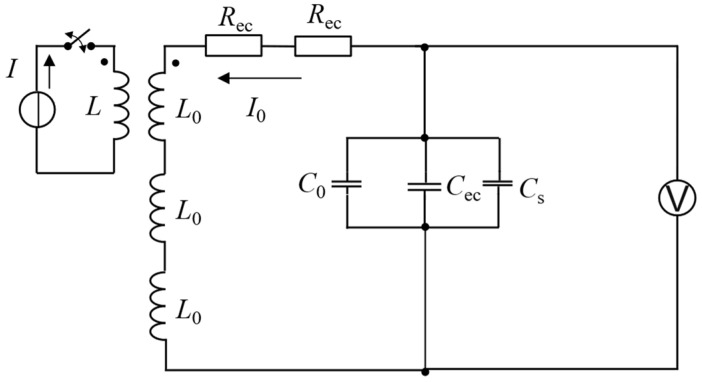
Equivalent electrical circuit for EMAT receiving process.

Therefore, the effect of the backplate position on the received signal can be investigated from the perspective of the coil-impedance variations. The receiving coil is made in the form of a printed circuit board, and the number of turns of the receiving coil is relatively large, compared with the transmitting coil. The analytical or FEM (finite element method) model of a receiving coil with the backplate is complicated. Hence, it would be very difficult to evaluate all the behaviors of the receiving process [13], and so the simplified equivalent circuit is selected to predict the behavior of a receiving coil with the backplate. For this purpose, the equivalent circuit model of the EMAT reception process is proposed, as shown in Figure 3, where *R*_0_, *L*_0_, and *C*_0_ are the self-resistance, the self-inductance, and the parasitic capacitance of the receiving coils, respectively. *R_ec_*, *L_ec_* and *C_ec_* are the reflected resistance, inductance, and capacitance, respectively, due to eddy currents produced in the metallic backplate. The magnitude of *R_ec_*, *L_ec_* and *C_ec_* would be affected by the gap between the backplate and the receiving coil, because they belong to the real and imaginary eddy-current impedance. In the case of the coil leads, *L_s_* and *C_s_* are the inductance and capacitance, respectively, of the cables for connecting the receiving coils. Due to coupling between the equivalent inductance of the receiving coil (*L*_0_) and the equivalent inductance of the specimen (*L*), the eddy currents (*I*) generated by the acoustic wave will produce induced current (*I*_0_) in the receiving coil.

According to the equivalent circuit in Figure 3, the equivalent component parameters satisfy the Equation (8)

(8) $$\begin{array}{l} R_{total}=R_{0}+R_{ec}\\ L_{total}=L_{0}+L_{ec}+L_{s}\\ C_{total}=C_{0}+C_{ec}+C_{s} \end{array}$$

where *C_total_* is the total capacitance of the equivalent circuit, *R_total_* is the total resistance, *L_total_* is the total inductance, *I*_0_ is the coil current, and, according to Kirchhoff’s voltage law, this can be obtained from the second-order ordinary differential equation in Equation (9) about *I*_0_.

(9) $$L_{total}\frac{dI_{0}}{dt}+R_{total}I_{0}+\frac{1}{C_{total}}\int I_{0}dt=0$$

The characteristic equation (auxiliary equation) is Equation (10)

(10) $$L_{total}C^{2}+R_{total}C_{total}+1=0$$

From Equation (10), the equivalent electrical circuit for the EMAT receiving process can be regarded as an oscillatory system. The damping coefficient *ζ*, the resonance angular frequency *ω*_0_, and the damped-oscillation angular-frequency *ω_d_* can be used to characterize the intrinsic properties of the system, which can be described as

(11) $$\zeta =\frac{R_{total}}{2L_{total}}$$

(12) $$\omega_{0}=\frac{1}{\sqrt{L_{total}C_{total}}}$$

(13) $$\omega_{d}=\sqrt{\omega_{0}^{2}-\zeta^{2}}$$

The capacitance, *C*_0_, is a parasitic parameter in the receiving coil of the EMAT, and it is commonly far smaller than the inductance, *L*_0_, depending on the condition of the underdamped circuit.

(14) $$I_{0}=\frac{U_{0}}{\omega_{d}L_{total}}e^{-\zeta t}\sin\left(\omega_{d}t\right)$$

In an underdamped equivalent circuit, the current will oscillate for multiple periods, and it will take some time to dissipate to a sufficiently small level.

### 2.2. Effects of the Backplate Position on Output Voltage Signal of EMAT

**Figure 4:**
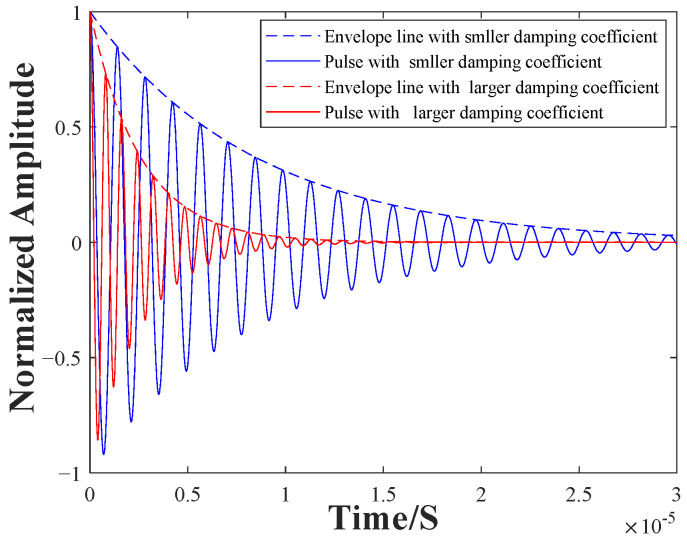
Simulation results of equivalent circuit model.

According to the establishment and analysis of the EMAT-receiving-process model above, it is clear that the extent of the interaction between the backplate and the receiving coil determines the total capacitance and inductance of the equivalent circuit, which further affects the damping coefficient *ζ* and damped-oscillation angular-frequency *ω_d_*. The pulses for different situations can be simulated using Equation (14). Figure 4 shows the simulated results with different damping coefficients. When the total inductance is larger, the damping coefficient is relatively small, and the damped-oscillation angular-frequency is relatively high. When the total inductance is smaller, the damping coefficient is relatively large, and the oscillating frequency is relatively low. At the same time, when the damping is rather large, the output current decays faster, and the oscillation duration is relatively short. This result may help to improve the resolution of the thickness measurement, because it can be used to reduce the pulsed width of the EMAT signal.

By means of simulation, it can be found that the variations of total inductance in the equivalent circuit can affect the pulsed width and frequency of the EMAT signal. This is owing to the fact that the equivalent elements of the receiving coil are affected by the vicinal backplate. The smaller the gap, the stronger the influence of the eddy currents in the backplate, the larger the *L_p_*, and the larger the corresponding *L_total_*. Hence, it can be inferred that the coil–backplate gap is a factor that must be considered during EMAT design, and the difference in the coil–backplate gap will be reflected in the amplitude response and damping coefficient of a single-pulse echo. Therefore, the position of the backplate is studied and then employed to optimize the characteristics of the EMAT signal.

## 3. Experiments and Results

The determination of the equivalent elements’ specific values of a particular receiving coil–backplate structure is required before the model presented above is used to predict the signal characteristics. The parameters in the equivalent circuit of the receiving coil–backplate structure with different gaps are obtained by means of impedance measurements and curve fitting. In order to verify the results predicted by the equivalent model, the signals of different EMAT configurations are also obtained by an experiment using the standard test block.

### 3.1. Experiment for Impedance Measurement of Models

**Figure 5:**
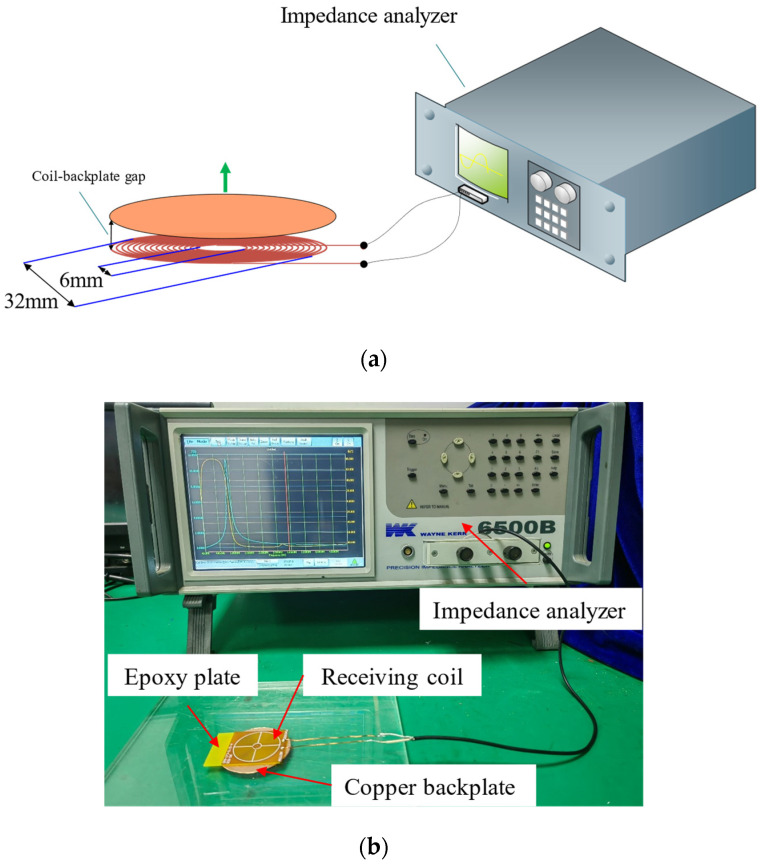
The impedance measurement platform. (**a**) Diagram of the experimental settings. (**b**) Photograph of the impedance measurement-platform.

**Figure 6:**
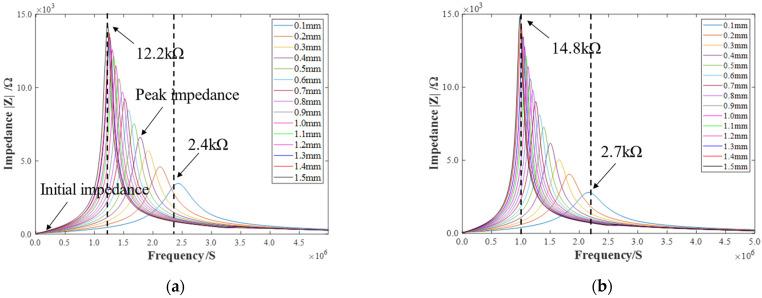
The impedance curves of (**a**) 153-turn receiving coil–backplate and (**b**) 173-turn receiving coil–backplate.

Figure 5 illustrates the configuration of the experimental setup used to obtain the specific coil impedance. In this experiment, two types of 4-layer coils with 153 and 173 turns, respectively, were measured. The outer diameter and inner diameter of the receiving coils were 32.0 mm and 6.0 mm, respectively. The backplate was a copper sheet with a diameter of 40.0 mm and a thickness of 0.3 mm. During measurement, the backplate was placed directly above the receiving coil. The gap between the copper backplate and the receiving coil ranged from 0.1 mm to 1.5 mm, with a step of 0.1 mm. The frequency of impedance measurement ranged from 20 Hz to 5 MHz, and each sweep acquired 800 points; this was followed by an averaging of the four times. Figure 6 shows the impedance curves measured by the impedance analyzer (WK 6500B). The impedance changes can be analyzed from the initial impedance and peak impedance. It can be noticed that the initial impedance is very close at different gaps; this is because the initial impedance is mainly the resistance value of the coil. However, the peak impedance-magnitude and frequency variations are significant, and it can be inferred that the inductance and capacitance have changed considerably. For a 153-turn coil, the peak magnitude of the impedance increases from 2.4 kΩ to 12.2 kΩ, and the peak frequency decreases from 2.4 MHz to 1.2 MHz when the receiving coil–backplate gap decreases from 1.5 mm to 0.1 mm. For a 175-turn coil, the peak magnitude of the impedance varies from 2.7 kΩ to 14.8 kΩ, and the frequency varies from 2.15 MHz to 0.99 MHz. At the same time, the variations of impedance are continuous with the coil–backplate gap for both coils, and the corresponding variations in inductance and capacitance are also continuous. The specific results are given in the next section of this paper.

### 3.2. Experiments for Output Signal Measurement

**Figure 7:**
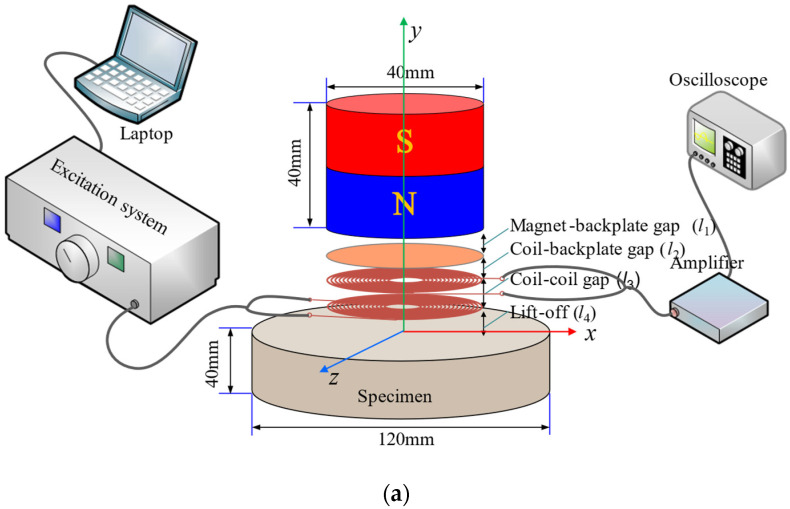

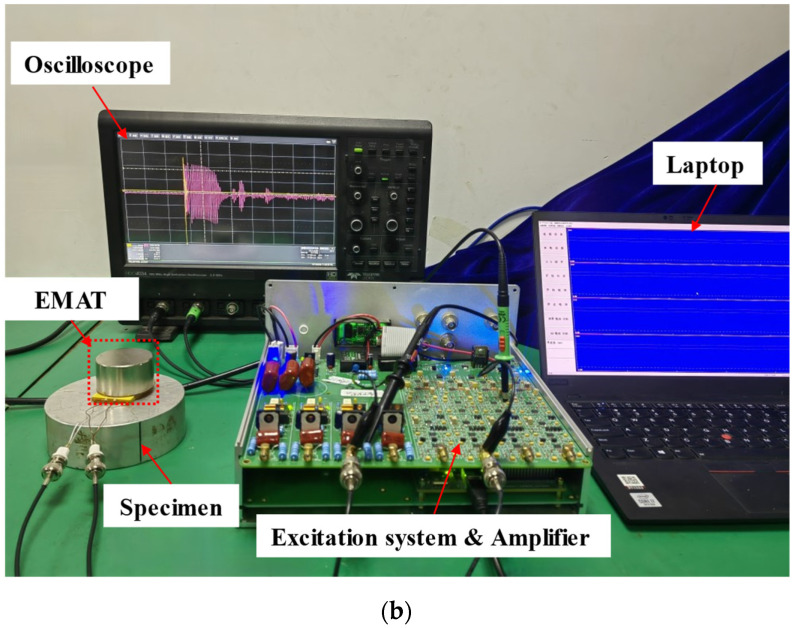
Experimental platform used to obtain the EMAT signals. (**a**) Diagram of the experimental settings. (**b**) Photograph of the experimental platform.

Figure 7 illustrates the configuration of the experimental setup used to obtain the EMAT signals. The transducer used in this experiment consisted of a transmitting coil with 16 turns, a receiving coil with 153/173 turns, a cylindrical magnet with a diameter of 40 mm and a height of 40 mm, and a copper backplate. The standard test block was an aluminum billet with a diameter of 120 mm and a thickness of 40 mm. In order to obtain the damping process of the whole pulse, a relatively thick block was selected for the experiment. In this study, the magnet–backplate gap was defined as *l*_1_, the receiving coil–copper-backplate gap was defined as *l*_2_, the transmitting coil-receiving coil gap was defined as *l*_3_, and the transmitting coil lift-off was defined as *l*_4_. The lift-off *l*_4_ was set to 3.00 mm, and the coil–coil gap *l*_3_ was set to 1.50 mm. The sum of *l*_2_ and *l*_1_ was 1.5 mm, and *l*_4_ and *l*_3_ were kept unchanged in the experiment, i.e., all the changes in the received signals can be attributed to the difference in the receiving coil–backplate gap. During the experiment, the transmitting coil was driven by a high-voltage pulse from the transmitting system, and the voltage signal in the receiving coil was acquired by an oscilloscope, after being amplified.

**Figure 8:**
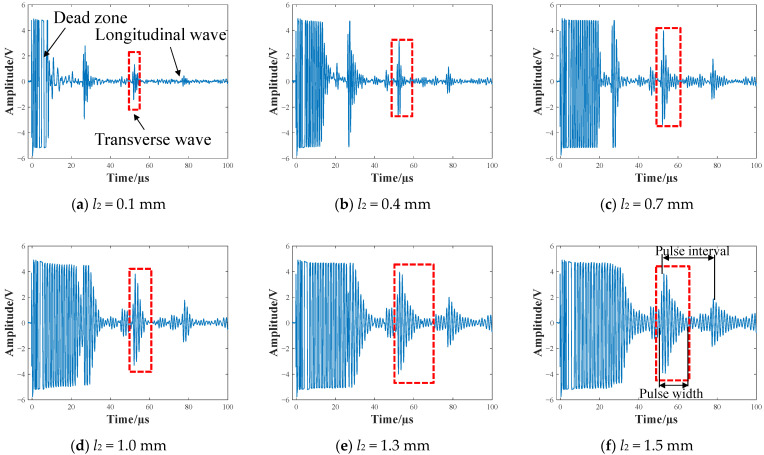
The signals of the 173-turn coil at the backplate position of (**a**) 0.1 mm, (**b**) 0.4 mm, (**c**) 0.7 mm, (**d**) 1.0 mm, (**e**) 1.3 mm, (**f**) 1.5 mm, respectively.

For brevity, Figure 8 only shows the thickness-measurement signal of the 173-turn coil with *l*_2_ = 0.1 mm, 0.4 mm, 0.7 mm, 1.0 mm, 1.3 mm, and 1.5 mm, respectively. A typical EMAT signal consists of the dead zone, the pulsed echoes, and the noise. The dead zone mainly comes from electromagnetic shocks. The greater the shock, the smaller the damping coefficient, and the longer the corresponding time duration of received pulses. It is shown that the dead zones of the signals become longer when the gap between the receiving coil and the copper backplate increases. Specifically, when the gap is 0.1 mm, the dead-zone length is less than 10 µs, and when the gap is 1.5 mm, the dead-zone length is more than 30 µs. This is also relevant to the increasing inductance and the decreasing damping coefficient of the EMAT. It is worth noting that, although the EMAT used is a transverse wave transducer, the transverse-to-longitudinal-conversion phenomenon is inevitable. Therefore, the transverse signals will be disturbed, causing some errors. Considering that the blocking zone may drown some of the pulse echoes after a change in the backplate position, the pulses between 50 µs and 60 µs were selected for comparison, marked by the red dashed box. The pulse width varies continuously with the spacing *l*_2_, as can be seen in Figure 8a–f. For the quantitative descriptions, the pulse width increased by 400% from 3 µs to 15 µs when the coil–plate gap (*l*_2_) increased from 0.1 mm to 1.5 mm. At the same time, the magnitude of the pulse amplitude increased by approximately 200% when *l*_2_ was 1.5 mm, compared to when *l*_2_ was 0.1 mm. Thus, the position of the backplate affects the amplitude and width of the pulse.

## 4. Results and Discussions

**Figure 9:**
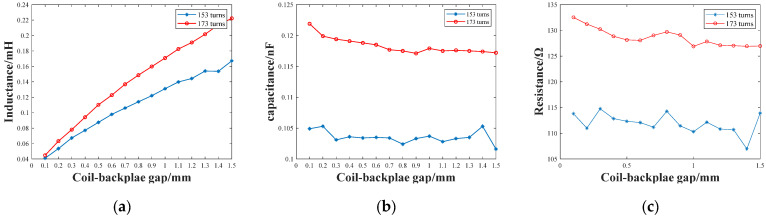
The total (**a**) resistance, (**b**) inductance, and (**c**) capacitance parameters of receiving coils, with the backplate at a different distance.

The impedance curves and signals of different receiving coil–backplate structures were obtained through the experiments in Section 3. To further investigate the relations between the parameters in the equivalent model and the signal characteristics when the relative positions of the backplate and the receiver coil are adjusted, the total resistance (*R_total_*), inductance (*L_total_*), and capacitance (*C_total_*) were first obtained by fitting the impedance curves using MATLAB. At this time, the inductance obtained is the sum of *L_c_*, *L_p_*, and *L_s_* in the equivalent circuit; the resistance is the sum of *R_c_* and *R_p_*; the capacitance is the sum of *C_c_*, *C_p_*, and *C_s_*. All the fitted results are shown in Figure 9. It is shown that all the parameters of the 173-turn coil are larger than those of the 153-turn coil, and the variations of the parameters in the 173-turn coil are more significant. In terms of the two receiving coils, the difference in resistance and capacitance are not obvious, and are less than 5% of the initial value for both coils. The value of the equivalent inductance increases significantly as the gap increases, with a gap of 1.5 mm being approximately 4~5.5 times that of 0.1 mm. Hence, the copper backplate has the most influence over the inductance in the coil. The damping coefficient and resonant frequency can be analyzed in conjunction with Equations (13) and (14).

**Figure 10:**
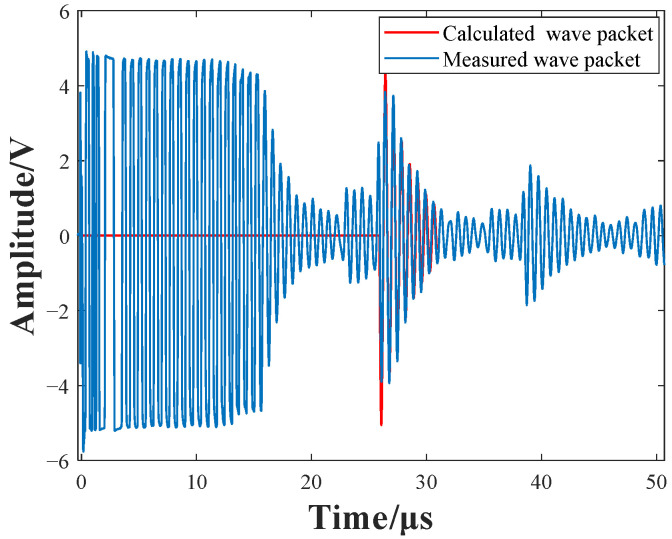
Theoretical calculation pulses and measurement-signals fitting results.

According to the impedance measurement results, the damping coefficient and self-resonant frequency of the receiving coil–backplate structure can be calculated by Equations (12) and (13) respectively. A theoretical pulse waveform can be obtained by Equation (15), which can then be fitted to the corresponding signal. Figure 10 shows one of the fitting results: the selected signal is measured with a 153-turn receiving coil, and the coil–backplate gap is 1.5 mm. In terms of pulse width and amplitude decay-rate over multiple periods, the theoretically calculated and measured wave packets are in good agreement. The calculated and measured frequency is 767 kHz and 745 kHz, respectively. The calculated and measured damping coefficient is 1.48 × 10^5^ and 1.59 × 10^5^, respectively.

**Figure 11:**
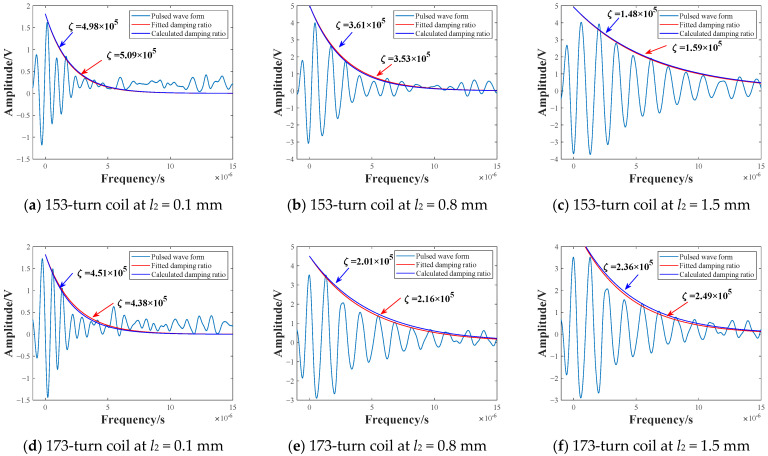
Fitting results of (**a**) 153-turn coil at *l*_2_ = 0.1 mm (**b**) 153-turn coil at *l*_2_ = 0.8 mm (**c**) 153-turn coil at *l*_2_ = 1.5 mm (**d**) 173-turn coil at *l*_2_ = 0.1 mm (**e**) 173-turn coil at *l*_2_ = 0.8 mm (**f**) 173-turn coil at *l*_2_ = 1.5 mm.

**Figure 12:**
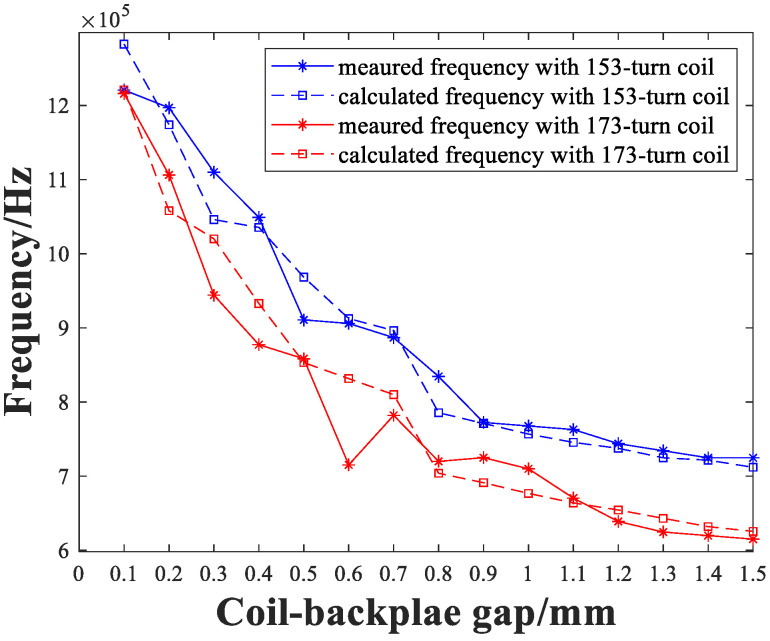
Measured and calculated frequencies of pulses with 153-turn coil and 173-turn coil.

Employing the approach stated above, the measurement results of the two different configuration receiving coils are verified. To focus on the effect of spacing on decay rate and pulse width, only envelope decay curves are drawn. The pulsed signals were selected when *l*_2_ was 0.1 mm, 0.8 mm and 1.5 mm, followed by fitting to the corresponding theoretically calculated damping-ratio, as shown in Figure 11. It can be seen that the signal-pulse width is consistent with the theoretical calculation, and the errors are less than 10%. Figure 12 shows the measured and calculated damped-oscillation angular-frequency of the selected pulsed wave-packets. It can be seen that the similarities between the results of the two different coils are good.

From the study above, the receiving coil–backplate gap has an impact on the frequencies and pulse width of the pulsed echoes in the signals. Moreover, the difference in signal amplitudes is also non-negligible, as can be seen from Figure 8. The initial amplitudes of the pulses also become large as the gap increases. The receiving coil couples the eddy currents in both the backplate and the specimen, and as the distance between the coil and the backplate increases, the coupling coefficient with the backplate decreases and, in contrast, the coupling coefficient with the specimen increases, with *L*_0_, *I*_0_ and *k* all increasing. It can be explained by the following equations

(15) $$U=\frac{k\sqrt{LL_{0}}}{\omega L_{0}}I\frac{1}{\omega C_{total}}$$

where *k* is the coefficient of coupling between the receiving coil and the specimen, *I* is the eddy current in the specimen, and *U* is the output voltage of the receiving coil.

**Figure 13:**
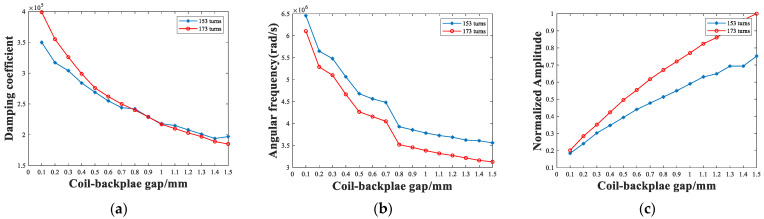
The (**a**) damping coefficients, (**b**) damped-oscillation angular-frequencies, and (**c**) amplitudes of EMATs with different receiving coil–backplate gaps.

Therefore, the selection of the backplate position needs to consider the damping coefficient, amplitude and frequency of the pulse, as shown in Figure 13. As the receiving coil–backplate gap increases, the influence of the backplate on the receiving coil diminishes, and the variation rate in all three parameters drops. The 173-turn coil has a larger damping coefficient than the 153-turn coil at a gap of less than 0.6 mm, and has a larger amplitude, despite the response frequency being 25% lower. Thus, for these two types of coils, a 173-turn with a gap of 0.5 mm or 0.6 mm would be appropriate. When the transducer is designed in conjunction with the thickness of the specimen, the backplate is required to be placed close to the coil for smaller thicknesses, to improve the attenuation of the pulse. For specimens with a large thickness, the backplate can be placed farther from the coil to improve the signal amplitude, but this may result in a larger magnet lift-off, and will require adjustment, in combination with further experiments.

## 5. Conclusions

The impacts of the backplate position on EMAT signals was studied. An equivalent circuit model of the receiving coil–backplate structure was established. Using this model, the effects of the size of the coil–backplate gap on the equivalent circuit model parameters can be predicted, including the initial amplitude, damping coefficient and response frequency. Thereby, the EMAT thickness measurement signal can be predicted, and the width and amplitude of the pulse can be modified as required, by adjusting the backplate position.

EMAT experiments were conducted to verify the above study, and it was proved that the backplate position has significant effects on the impedance of the receiving coil and the characteristics of the signal. For the two configurations of coils, when the coil–backplate gap was increased in the range of 0.1 mm to 1.5 mm, the corresponding inductance was increased by up to 400%, while the resistance and capacitance were decreased by approximately 10%. In this process, the signal amplitude and pulse width were increased, while the damping coefficient and resonant frequency were decreased, and the exact amount of these changes depended on the configurations of the coils.

## Data Availability

Not applicable.
